# Effects of different pollination methods on tomato fruits’ quality and metabolism

**DOI:** 10.3389/fpls.2025.1560186

**Published:** 2025-04-04

**Authors:** Wei-Hua Ma, Wen-Qin Wu, Huai-Lei Song, Jia Lei, Li-Xin Li

**Affiliations:** College of Horticulture, Shanxi Agricultural University, Taiyuan, China

**Keywords:** tomato, honeybee, bumblebee, plant growth regulator, quality, endogenous hormones content, metabolomics

## Abstract

Bee pollination can affect tomato yield and quality. The mechanism of improving the yield and quality of tomatoes by bee pollination is not clear, and few studies have been conducted. To understand how bee pollination affects tomato quality, by using respectively weighing, vernier caliper, handheld refractometer, pH meter to measure single fruit weight, fruit size, the sugar content, and the pH value, enzyme linked immunosorbent assay (ELISA) to determine endogenous hormone content, and LC-MS to perform untargeted metabolomics analysis, we compared these physiological indicators, endogenous hormone levels, and metabolism of tomato fruits pollinated after honeybee, bumblebee, and plan growth regulator (PGR) pollination. Our results indicate that the tomatoes pollinated by bumblebees were heavier and larger than those pollinated by honeybees and PGR. The sugar content of tomatoes pollinated by honeybees and bumblebees significantly respectively increased by 7.96% and 10.18% than that of tomatoes pollinated by PGR. The pH value of tomatoes pollinated by honeybees (3.99 ± 0.02) and bumblebees (3.94 ± 0.03) was significantly lower than that of tomatoes pollinated by PGR (4.19 ± 0.04) (p < 0.05). Different pollination methods significantly affected the content of endogenous hormones in fruits. In five endogenous hormones, the highest content was gibberellin (GA) in honeybee pollination treatment, IAA in bumblebee treatment, and the highest contents were abscisic acid (ABA), zeatin (ZT), and *N* ^6^-(Δ^2^-isopentenyl) adenosine (iPA) in PGR treatment. It is speculated that different pollination methods may regulate the maturity and quality of tomatoes through different hormone levels. There were respectively five different metabolites (three upregulated and two downregulated), 95 different metabolites (59 upregulated and 36 downregulated), and 95 different metabolites (56 upregulated and 39 downregulated) in honeybee pollination vs. bumblebee pollination, honeybee pollination vs. PGR pollination, and bumblebee pollination vs. PGR pollination. Metabolites are mainly involved in phenylpropanoid biosynthesis, flavonoid biosynthesis pathway, and stilbenoid, diarylheptanoid and gingerol biosynthesis. Compared with PGR pollination, the metabolism of amino acids, vitamins, sugars, flavor substances, and organic acids with antioxidant physiological effects in honeybee pollination and bumblebee pollination groups was significantly higher. It can be inferred that the tomato fruit after bee pollination may have a better taste and is favorable to resisting diseases. These results provide valuable insight for uncovering the mechanism of how bee pollination enhances tomato fruit flavor and will enhance our understanding of interactions between bee pollinators and fruit development processes.

## 1 Introduction

Pollination is vital for the reproduction of plants and improves the quantity and quality of crops. Pollination is a complex process for plants, and the effect of pollination is closely related to the morphology of flowers, nectar secretion, pollens, and pollinators (Balthazar et al., 2025; Chartier et al., 2025). Bees, as the most important pollinators, are responsible for over 90% of visits to the flowers of the most common crops and wild flowering plant species and are of great significance to sustaining healthy ecosystems and ensuring food security (Requier et al., 2023; Schleimer and Frantz, 2025; Aristizábal et al., 2025; Chartier et al., 2025). Bee pollination can improve the yield and quality of crops (fruit setting, fruit weight, size, malformations, firmness, etc.), influence crop nutritional and commercial value [sugar, acidity, vitamin C content (Vc), volatile organic compounds (VOCs), commercial grade, shelf life, etc.] (Georg et al., 2012; Bartomeus et al., 2014; Klatt et al., 2013; Bashir et al., 2018; Nicholson and Ricketts, 2019; Zhang et al., 2022; Magalhães et al., 2024; Aristizábal et al., 2025), and even promote rapid divergent evolution in plant growth (Dorey and Schiestl, 2024). Currently, the bee species used for pollination include honeybees, bumblebees, stingless bees (Cooley and Vallejo-Marín, 2021), and wild bees (Greenleaf and Kremen, 2006). The crops involved in bee pollination include strawberries, blueberries, watermelons, melons, coffee, gherkin, and tomatoes.

As the second most common and important commercial fruit or vegetable with approximately 182.3 million tons of tomato fruits on 4.85 million ha each year, tomato is one of the crops that have been more studied for bee pollination (Quinet et al., 2019). At present, bumblebee pollination is the common pollination method for tomatoes (Morandin et al., 2001a, b; Strange, 2015; Cooley and Vallejo-Marín, 2021; Zhang et al., 2022), and honeybees (Sabara and Winston, 2003; Sabara et al., 2004; Higo et al., 2004) and stingless bees (Santos et al., 2009; Wongsa et al., 2023) are also used for tomato pollination. Bee pollination can affect tomato fruit set, fruit weight, diameter, shape, seed number, sugar content, acidity, Vc, and VOCs; decrease cavities, malformed fruits, etc.; and reduce labor costs; it is welcomed by many growers and the majority of producers (Morandin et al., 2001a, b; Sabara and Winston, 2003; Sabara et al., 2004; Higo et al., 2004; Bell et al., 2006; Palma et al., 2008; Santos et al., 2009; Strange, 2015; Nishimura, 2021; Zhang et al., 2022; Wongsa et al., 2023).

However, there is little research on the mechanism of the effect of bee pollination on tomato fruit quality, which is also mentioned in coffee bee pollination (Aristizábal et al., 2025). Some studies have been about the relationship between pollination and crop quality in terms of pollen tube growth, pollen quantity, stigma, fertilization, etc (Stavert et al., 2020; Balthazar et al., 2025; Chartier et al., 2025). Phytohormones are important regulatory substances in fruit development and maturation, and metabolites (Srivastava and Handa, 2005; Quinet et al., 2019; Fenn and Giovannoni, 2021; Jia et al., 2024). Untargeted metabolomics analysis can reveal the bioactive phytochemicals of fruits (Hu et al., 2023). Therefore, we assume that the content of endogenous hormones in tomatoes pollinated by bees is different from that in tomatoes pollinated by plant growth regulator (PGR), which in turn affects the quality and maturity of tomatoes; the metabolites of tomatoes pollinated by bees are richer than those of tomatoes pollinated by PGR, and the taste and flavor of tomatoes pollinated by bees are better. Because of the advantages of locality, large population, convenient breeding and management, easy accessibility, and convenient transportation, honeybees have always been the main force of greenhouse crop pollination (Rucker et al., 2012) and allow the needs of tomato production to be met (Sabara et al., 2004). Since the commercialization degree of stingless bees is not high, and the adaptability in the local area also needs to be tested, stingless bees are not used in the study. In addition to bee pollination, spraying PGR (Mousawinejad et al., 2014) is also commonly used for greenhouse tomato pollination. Therefore, we used honeybees, bumblebees, and PGR to pollinate greenhouse tomatoes; then compared the physiological indicators, endogenous hormones level, and metabolism of pollinated tomatoes; and analyzed the metabolic differences of different pollination methods to verify the above assumption. Based on these results, it is expected to provide theoretical data for revealing bee pollination mechanisms on tomato flavor.

## 2 Materials and methods

### 2.1 Tomato, insect, and pollination

The experiment was conducted from May to August 2020 at the greenhouse tomato planting base in Dabai Village, Taigu, Shanxi, with each greenhouse having a size of 110 m × 8 m × 3 m. The tomato variety was Vienna 2. Three greenhouses with the same conditions were used for three treatments: honeybee pollination (T1), bumblebee pollination (T2), and plant growth regulator pollination (T3). Each greenhouse was divided into three plots as three replicates. Honeybees (*Apis mellifera ligustrica*) with three combs (6,000–7,500 honeybees) were from the experimental apiary of Shanxi Agricultural University. Bumblebees (*Bombus terrestris*) (80–100 bumblebees) were bought from the Woofuntech Bio-Control Company (Hebei, China). The plant growth regulator (forchlorfenuron or CPPU) (Anshan Huaxin Agricultural Technology Co., Ltd., Anshan, China) was sprayed according to instructions on sunny mornings.

### 2.2 Measuring fruit characteristics

A total of 60 flowers (20 flowers on each plot) on each treatment were labeled immediately after pollination. At 20 days, the fruit-setting rate was counted. Fruit-setting rate (%) = (the number of fruits) * 100/the number of flowers labeled.

After fruit ripening, 10 fruits as one replicate were randomly selected from each plot; in total, 30 fruits in each treatment were collected to test the single fruit weight and the transverse and longitudinal diameters. Each fruit was weighed, and the transverse and longitudinal diameters were measured using vernier caliper. Then, the sugar content was measured using a handheld refractometer, and the pH of 15 fruits in each treatment was measured using a pH meter.

These aforementioned parameters were expressed as means ± SE. These parameters were compared using one-way ANOVA and Tukey’s test. The significance level was established as p < 0.05. The statistical analyses were made using the GraphPad Prism 5 software. In the figures, *, **, and *** indicate significant differences between the two treatments (p < 0.05, p < 0.01, and p < 0.001, respectively), and ns indicates that there was no significant difference between the two treatments (p > 0.05).

### 2.3 Determination of endogenous hormone content

After sugar content and pH value were measured, five fruits of each treatment were chopped and combined as a replicate, with 15 fruits for three replicates, and then frozen at −80°C for the detection of endogenous hormones and metabolome.

Indole-3-acetic acid (IAA), abscisic acid (ABA), gibberellin (GA), zeatin (ZT), and *N* ^6^-(Δ^2^-isopentenyl)adenosine (iPA) contents were detected using corresponding ELISA kits. Data analysis was the same as in Section 2.2.

### 2.4 Untargeted metabolomics analysis

An evenly mixed sample measuring 100 mg was taken, placed in a 2-mL centrifuge tube, added with 1 mL 70% methanol and a 3-mm steel ball, shaken and crushed for 3 min using a grinding instrument (JXFSTPRP-48, 70 Hz), and then underwent ultrasound at low temperature (40 kHz) for 10 min after cooling. It was centrifuged at a speed of 12,000 rpm for 10 min at 4°C. The supernatant was taken, diluted 2–100 times, and then tested on a 0.22-μm polytetrafluoroethylene (PTFE) filter head.

The LC-MS (Thermo, Waltham, MA, USA; UltiMate 3000 LC, Q Active HF) analysis platform was used for metabolite analysis, and the chromatographic column was Zorbax Eclipse C18 (1.8 μm, 2.1 mm * 100 mm). The chromatographic separation conditions were as follows: column temperature of 30°C and flow rate of 0.3 mL/min. Mobile phase A was composed of 0.1% formic acid, and B was composed of acetonitrile. The mobile phases consisted of 0.1% formic acid aqueous solution (A) and acetonitrile solution (B) for gradient elution (0–2 min, 5% B; 2–7 min, 30% B; 7–14 min, 78% B; 14–20 min, 95% B; 20–25 min, 5% B), with an injection volume of 2 μL. The temperature of the automatic sampler was 4°C.

High-resolution Q-Exactive HF mass spectrometry was used for detection in positive and negative ion modes of electrospray ionization (ESI). The ionization mode was heated electrospray ionization (HESI). The mass spectrometry scanning range was from 100 to 1,500 m/z, and the scanning speed was 1,000 Da/s. The spray voltage was 3.50 kV. The capillary temperature (ion transfer tube) was 330°C. The heater temperature was 325°C. Sheath gas, auxiliary gas, and purge gas were 50.0, 12.0, and 1.0 orbital units, respectively. Nitrogen was used in spray-stabilized high-energy collision-induced dissociation (HCD) batteries. The calibration solution was used for instrument calibration, and analysis was conducted in full scan mode (FM).

The Thermo mzCloud online database, Thermo mzValut local database, ChemSpider database, etc., were used for the identification and quantitative analysis of metabolites. Differential expression multiple analysis, principal component analysis (PCA), orthogonal projections to latent structures–discriminant analysis (OPLS-DA) (SIMCA-P V14.1, MKS Data Analytics Solutions, Umea, Sweden), and other analytical methods were used to analyze the metabolomics results so as to obtain the differential metabolites between groups. The metabolites with fold change (FC) ≥ 2 and FC ≤ 0.5, p < 0.05, and variable importance in the projection (VIP) ≥ 1 (the projected importance value of a variable in the partial least squares model) were selected as the differential metabolites. The Kyoto Encyclopedia of Genes and Genomes (KEGG) database was used to annotate the differential metabolites. The annotation results of KEGG metabolites with significant differences were classified according to the types of KEGG pathways, and MetaboAnalystR was used to enrich KEGG pathways.

## 3 Results

### 3.1 The fruit-setting rate and fruit quality

**Figure 1:**
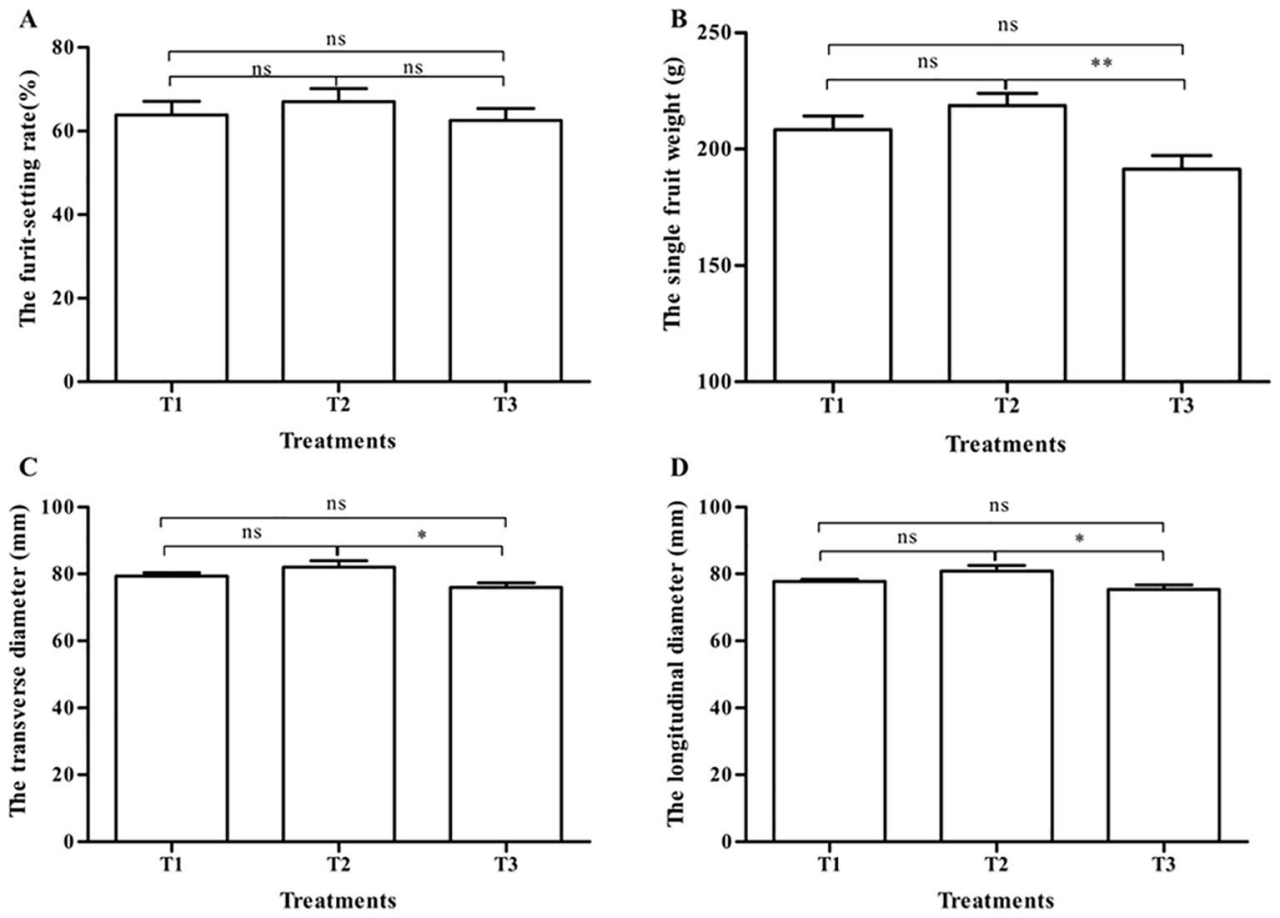
Tomato fruit-setting rate and appearance quality under three pollination methods. **(A)** The fruit-setting rate. **(B)** The single fruit weight. **(C)** The transverse diameter. **(D)** The longitudinal diameter. Note: T1, honeybee pollination; T2, bumblebee pollination; T3, PGR pollination. * and ** respectively indicate significant difference between the two treatments (p < 0.05 and p < 0.01). ns indicates that no significant difference between the two treatments (p > 0.05). Datas were shown as mean ± SE. The same as below. PGR, plant growth regulator.

Tomato appearance quality under three pollination methods is shown in **Figure 1**. There was no significant difference in the fruit-setting rate among the three treatments (p > 0.05) (**Figure 1A**). The weight of a single fruit after bumblebee pollination (T2) was 218.6 ± 5.2 g, the highest in the three treatments, which had obviously significant differences in contrast to PGR pollination (191.5 ± 5.7 g) (T3) (p < 0.01). There were no significant differences (p > 0.05) between T1 and T2 and between T1 and T3 (**Figure 1B**). In the transverse diameter and the longitudinal diameter, the fruits in T1 and T2 were both larger than those in T3, and the T2 group fruits were significantly larger than the T3 group fruits (p < 0.05).

**Figure 2:**
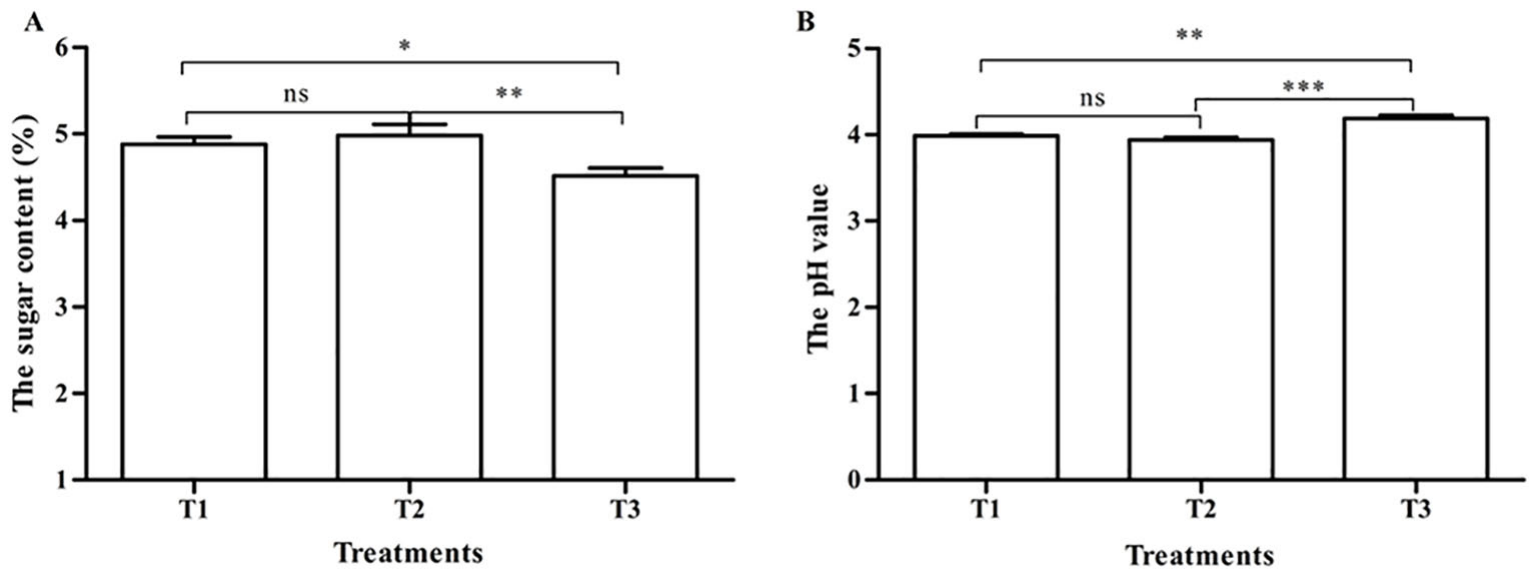
The sugar content and pH value in tomato fruits under three pollination methods. **(A)** The sugar content. **(B)** The pH value. Note: *, **, and *** respectively indicate significant difference between the two treatments (p < 0.05, p < 0.01, and p < 0.001). ns indicates that no significantly different between the two treatments (P>0.05). The same as below.

The sugar content and pH value of the three treatments are shown in **Figure 2**. The sugar content of tomatoes pollinated by honeybees (4.88 ± 0.08) and bumblebees (4.98 ± 0.13) was significantly higher than that of tomatoes pollinated by PGR (4.52 ± 0.09) (p < 0.05) (**Figure 2A**). The pH value of tomatoes pollinated by honeybees (3.99 ± 0.02) and bumblebees (3.94 ± 0.03) was significantly lower than that of tomatoes pollinated by PGR (4.19 ± 0.04) (p < 0.05) (**Figure 2B**). There was no significant difference in the sugar content and pH value of tomatoes pollinated by honeybees and bumblebees (p > 0.05) (**Figures 2A, B**).

### 3.2 The content of endogenous hormones

**Figure 3:**
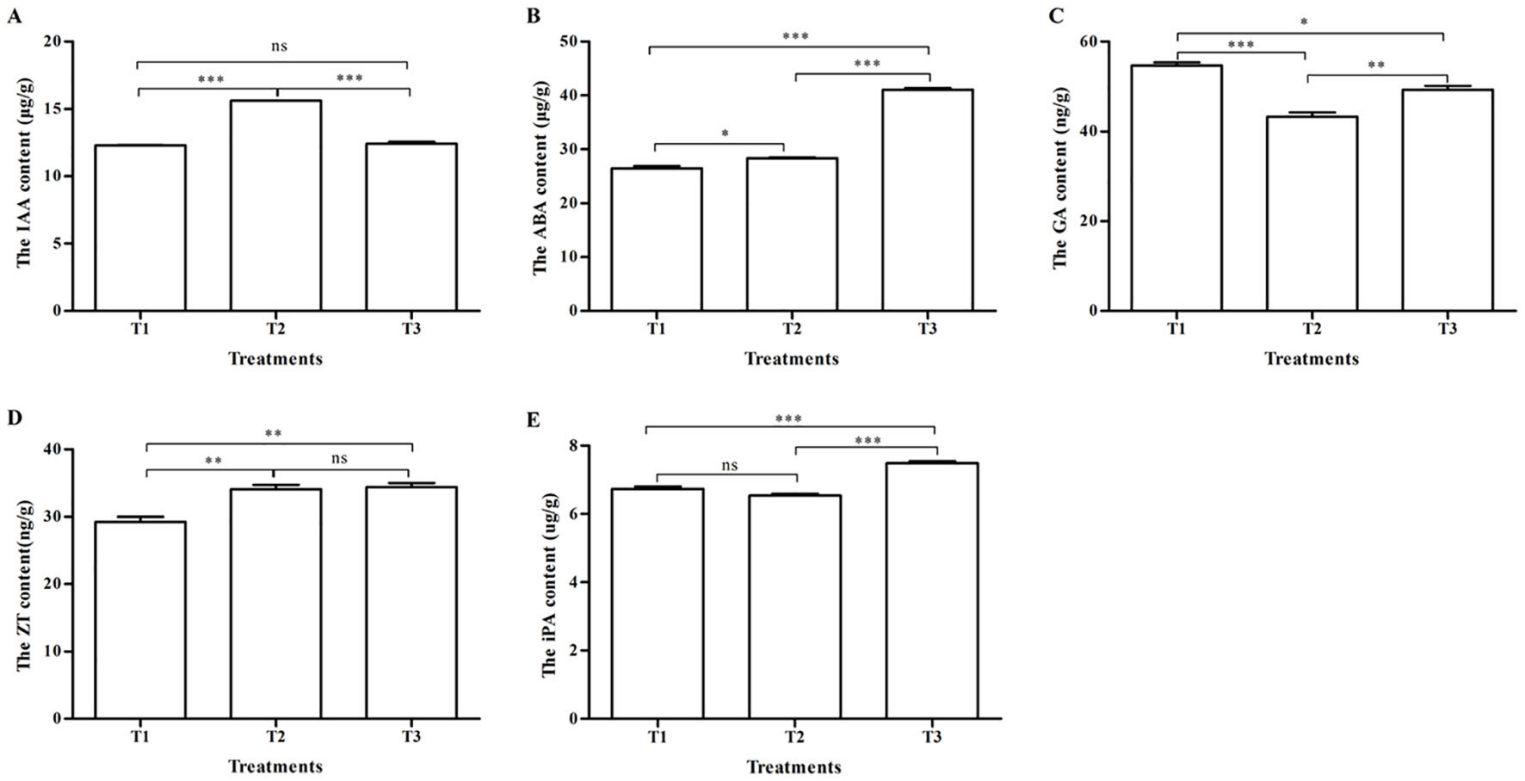
The contents of endogenous hormones in tomatoes under three pollination methods. **(A)** The IAA content. **(B)** The ABA content. **(C)** The GA content. **(D)** The ZT content. **(E)** The iPA content. Note: IAA, indole-3-acetic acid; ABA, abscisic acid; GA, gibberellin; ZT, zeatin; iPA, *N* ^6^-(Δ^2^-isopentenyl)adenosine. *, **, and *** respectively indicates that significantly different between the two treatments (P<0.05, P<0.01 and P<0.001). ns indicates that no significantly different between the two treatments (P>0.05).

Under different pollination methods, there was a significant difference in endogenous hormone content in tomatoes (p < 0.05) (**Figure 3**). The IAA content after pollination by bumblebees (T2) was 15.62 ± 0.01 μg/g, the highest in the three treatments, and has obvious significant differences in contrast to honeybee pollination (T1) and PGR pollination (T3) (p < 0.001) (**Figure 3A**). There were no significant differences (p > 0.05) between T1 and T3 (**Figure 3A**). The ABA content in T1, T2, and T3 are shown in **Figure 3B**, which were respectively 26.26 ± 0.46, 28.35 ± 0.13, and 41.08 ± 0.33 μg/g. There were significant differences between T1, T2, and T3 (p < 0.001) and between T1 and T2 (p < 0.05). As regards the GA content (**Figure 3C**), there were significant differences between T1 (54.68 ± 0.73 ng/g) and T2 (43.26 ± 0.98 ng/g) (p < 0.001), T1 and T3 (49.31 ± 0.87 ng/g) (p < 0.05), and T2 and T3 (p < 0.01). The ZT content of T1, T2, and T3 were respectively 29.25 ± 0.766, 34.12 ± 0.623, and 34.4 ± 0.630 ng/g (**Figure 3D**). There were significant differences between T1 and T2 and between T1 and T3 (p < 0.01). The iPA content of three treatments in descending order was T3 (7.487 ± 0.057 μg/g), T1 (6.739 ± 0.067 μg/g), and T2 (6.535 ± 0.062 μg/g). There were significant differences between T1 and T3 and between T2 and T3 (p < 0.001) (**Figure 3E**).

### 3.3 Metabolomics analysis

#### 3.3.1 PCA

After analyzing the tomato metabolites under three different pollination methods, among all the detected metabolites, 639 positive patterns and 749 negative patterns matched the substances in the database (**Supplementary File 1**). The PCA of all samples showed good clustering of internal Quality Control (QC) samples, which confirms excellent instrumental stability during sample analysis (**Supplementary File 2**).

**Figure 4:**
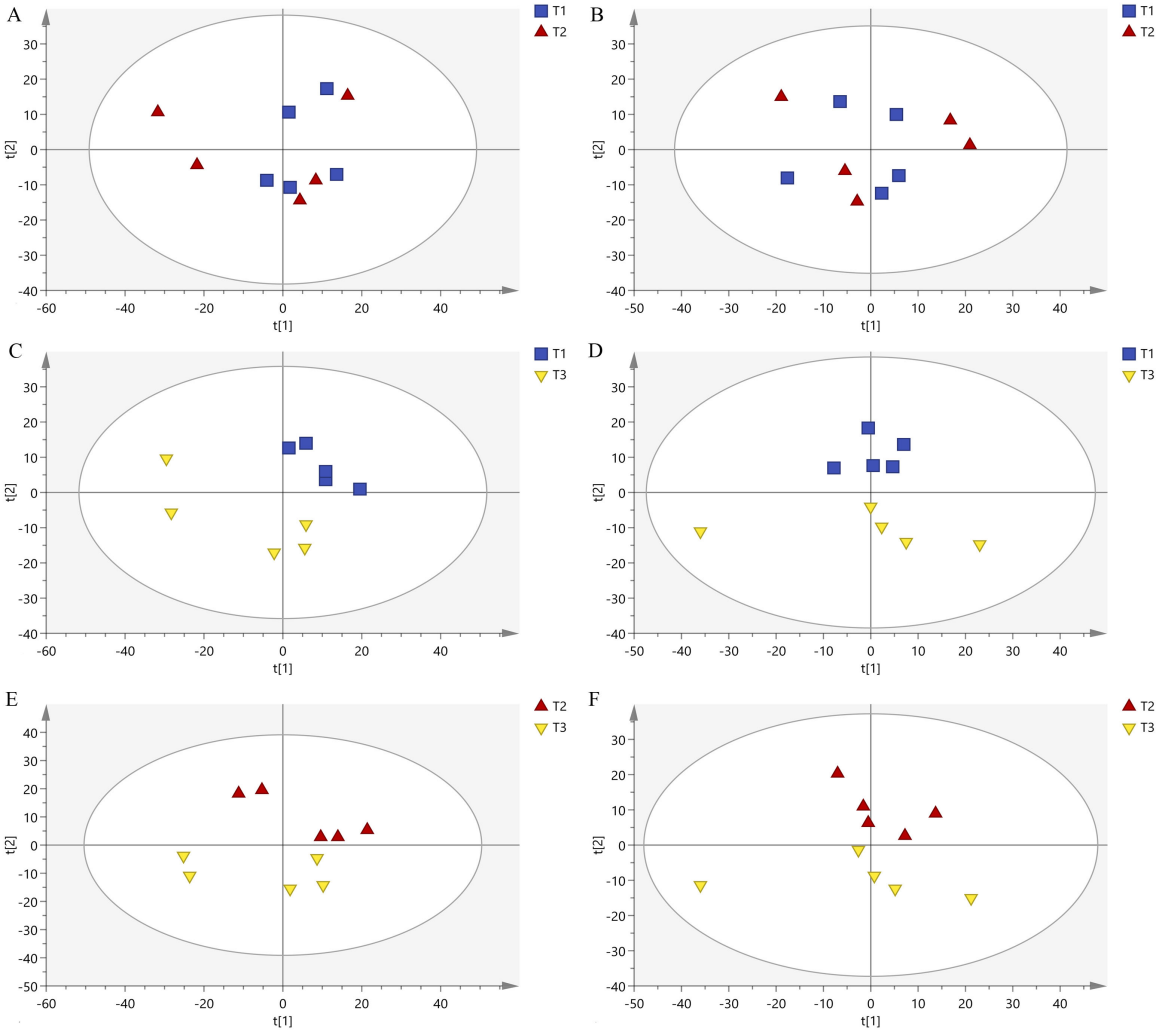
PCA diagram in negative ion mode and positive ion mode among three treatments: **(A)** T1 vs. T2 (negative mode), **(B)** T1 vs. T2 (positive mode), **(C)** T1 vs. T3 (negative mode), **(D)** T1 vs. T3 (positive mode), **(E)** T2 vs. T3 (negative mode), and **(F)** T2 vs. T3 (positive mode).

The PCA results of samples under three different pollination methods were shown in **Figure 4** and **Supplementary File 3**. The samples in the honeybee pollination and bumble pollination had more overlap (**Figures 4A, B**), indicating that the two pollination methods had partly common effects on tomato metabolites. The distribution areas of the samples in the honeybee pollination and bumblebee pollination were different from those in PGR pollination, and they can be completely separated in space (**Figures 4C–F**). There was a significant difference in the chemical composition of the samples pollinated by bees and those pollinated by PGR.

#### 3.3.2 Different metabolites

The metabolite content of samples from different treatments was compared in pairs using the OPLS-DA model. The OPLS-DA model parameters and verification are shown in **Supplementary Files 4**, **5**.

After screening and identification, different metabolites were obtained in all groups under different modes, as shown in **Table 1**. In general, the different metabolites of T1 vs. T2 were few, with only five metabolic differentials in total, while the metabolites of T1 vs. T3 and T2 vs. T3 were different greatly (**Table 1**). Both T1 vs. T3 and T2 vs. T3 had 95 different metabolites, of which 69 were repeated (**Table 1**; **Supplementary File 6**). These results indicate that honeybee pollination and bumblebee pollination had basically the same effects on tomato metabolites. Compared with PGR pollination, the effects of the two on tomato metabolites were significantly different.

**Table 1:** The number of different metabolites between different treatments.

Ion mode	The number of different metabolites		
	Up	Down	Total
T1vsT2_diff_neg	0	1	1
T1vsT2_diff_pos	3	1	4
T1vsT3_diff_neg	22	25	47
T1vsT3_diff_pos	37	11	48
T2vsT3_diff_neg	30	24	54
T2vsT3_diff_pos	26	15	41

One metabolite (Flavonol base + 4O, O-Hex-dHex-Pen) was downregulated in the T1 vs. T2-neg mode (p < 0.05) (**Table 1**, **Supplementary File 6**). In the T1 vs. T2-pos mode, one metabolite (Acetamiprid) was downregulated and three metabolites were upregulated (p < 0.05) (**Table 1**, **Supplementary File 6**). A total of 47 metabolites were significantly different in the T1 vs. T3-neg mode (p < 0.05), among which 22 metabolites were upregulated and 25 metabolites were downregulated (**Table 1**). A total of 48 metabolites were significantly different in the T1 vs. T3-pos mode (p < 0.05), among which 37 metabolites were upregulated and 11 metabolites were downregulated (**Table 1**). A total of 54 metabolites were significantly different in the T2 vs. T3-neg mode (p < 0.05), of which 30 metabolites were upregulated and 24 metabolites were downregulated (**Table 1**). A total of 41 metabolites were significantly different in the T1 vs. T3-pos mode (p < 0.05), among which 26 metabolites were upregulated and 15 metabolites were downregulated (**Table 1**).

In terms of the types of differential metabolites, it mainly included several categories: amino acid derivatives and dipeptides, vitamins, sugars and their derivatives, nucleic acids and their derivatives, flavor substances, organic acids, etc. The contents of organic acids, sugars, amino acids, and peptides in bee pollination were significantly different from those in PGR pollination. It is worth noting that there were many bioactive substances in differential metabolites, such as neochlorogenic acid, caffeic acid, and chlorogenic acid, which have antioxidant functions. These substances were highly metabolized in T1 and T2, significantly higher than in T3 (**Supplementary File 6**).

#### 3.3.3 KEGG analysis of differential metabolites

**Figure 5:**
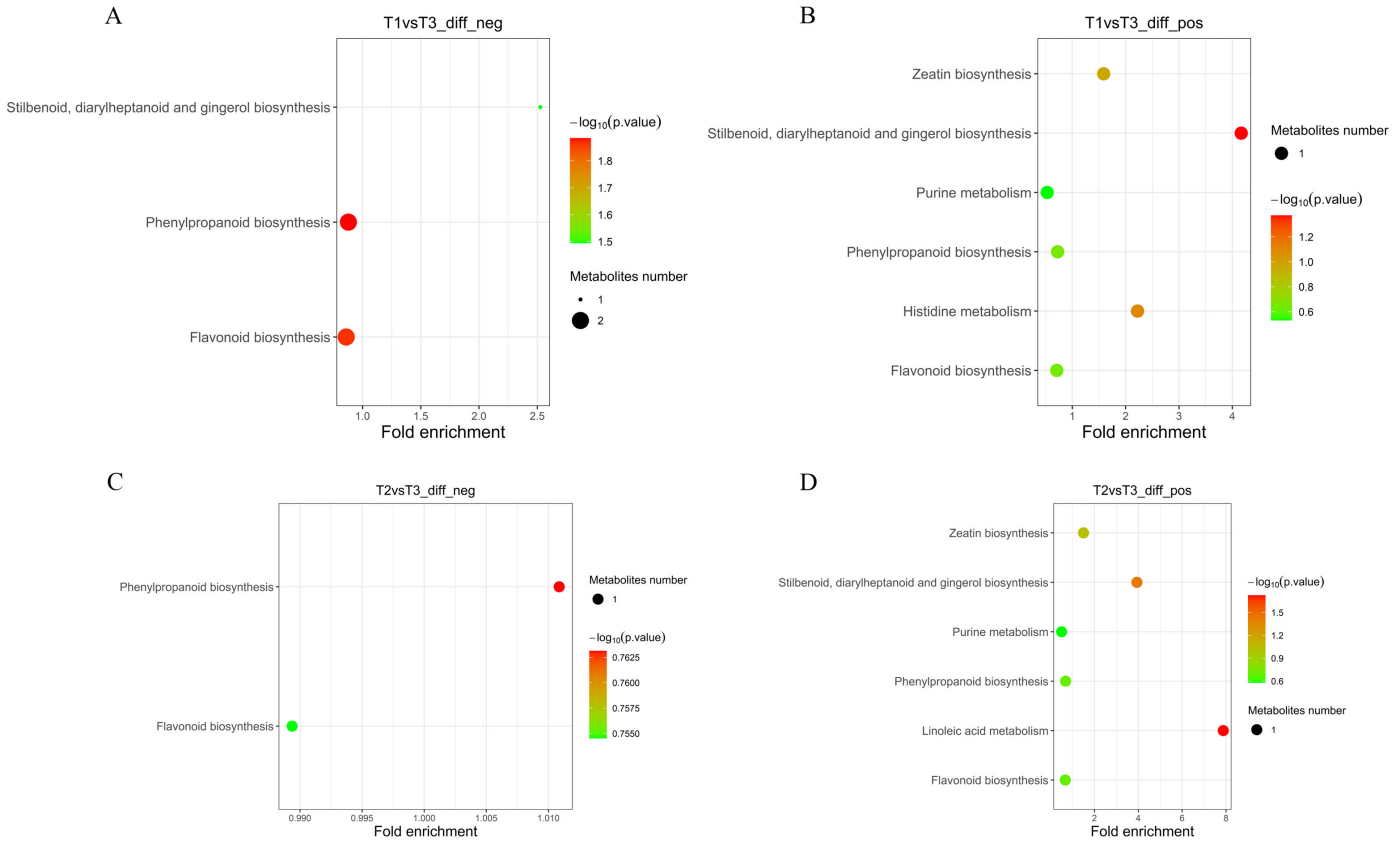
KEGG enrichment maps of differential metabolites under negative and positive ion modes. **(A)** T1 vs T3 (negative mode), **(B)** T1 vs T3 (positive mode), **(C)** T2 vs T3 (negative mode), **(D)** T2 vs T3 (positive mode).

Different metabolites were mapped to the KEGG database, and the annotation results of the significantly different metabolites KEGG were classified according to the pathway type in KEGG (**Supplementary File 7**). Further enrichment analysis of the KEGG pathway was performed. In the T1 vs. T3-neg mode, different metabolites were significantly enriched in phenylpropanoid biosynthesis and flavonoid biosynthesis (**Figure 5A**). Chlorogenic acid was highly metabolized in all two pathways. Ferulic acid was highly metabolized in the phenylpropanoid biosynthesis pathway. Naringenin was highly metabolized in the flavonoid biosynthesis pathway (**Supplementary File 7**). Stilbenoid, diarylheptanoid and gingerol biosynthesis, histidine metabolism, and zeatin biosynthesis were significantly enriched in the T1 vs. T3-pos mode (**Figure 5B**). Adenosine 5′-monophosphate was highly metabolized in zeatin biosynthesis. Histamine metabolism declines in the histidine metabolism pathway. Chlorogenic acid in the stilbenoid, diarylheptanoid and gingerol biosynthesis, phenylpropanoid biosynthesis, and flavonoid biosynthesis pathways were metabolically exuberant. Coumarin and umbelliferone in the phenylpropanoid biosynthesis pathways were metabolically exuberant (**Supplementary File 7**).

In the T2 vs. T3-neg mode (**Figure 5C**), the phenylpropanoid biosynthesis pathway and flavonoid biosynthesis pathway were significantly enrichment. Ferulic acid metabolism in the phenylpropanoid biosynthesis pathway was strong. Naringenin metabolism was exuberant in the flavonoid biosynthesis pathway. Linoleic acid metabolism, stilbenoid, diarylheptanoid and gingerol biosynthesis, and zeatin biosynthesis were significantly enriched in the T2 vs. T3-pos mode (**Figure 5D**). For the T2 vs. T3-pos mode, chlorogenic acid in stilbenoid, diarylheptanoid and gingerol biosynthesis, phenylpropanoid biosynthesis, and flavonoid biosynthesis pathway was highly metabolized (**Figure 5D**). Adenosine 5′-monophosphate was highly metabolized in zeatin biosynthesis. 13-HPODE was strongly downregulated in the linoleic acid metabolism pathway. Umbelliferone and coumarin were highly metabolized in the phenylpropanoid biosynthesis pathway (**Supplementary File 7**).

The metabolites of T1 vs. T2 were enriched, but different metabolites could not be enriched to the metabolic pathway, which may be due to the difference of metabolites that did not have the corresponding KEGG pathway.

## 4 Discussion

Bee pollination can affect tomato yield and quality (nutrients and flavor) (Sabara and Winston, 2003; Cooley and Vallejo-Marín, 2021), especially bumblebees (Zhang et al., 2022; Wongsa et al., 2023). Our results showed that the fruit-setting rate of tomatoes could meet the production needs after both bee pollination and PGR pollination. This is also consistent with the three methods of pollination commonly used to improve fruit set in production (Sabara et al., 2004; Mousawinejad et al., 2014; Zhang et al., 2022). The literature shows that the fruit-setting rate of bumblebee pollination is significantly higher than that of PGR (Zhang et al., 2022), which is inconsistent with this study and may be related to the different local tomato varieties, cultivation management, and other conditions. In fruit weight and size, bumblebee pollination significantly increased the single fruit weight and fruit size than honeybee pollination and PGR pollination. These are consistent with the results of several studies (Strange, 2015; Al-Attal et al., 2003; Cooley and Vallejo-Marín, 2021; Zhang et al., 2022; Huang et al., 2024). There was no significant difference between honeybee pollination and PGR pollination in increasing the weight and size of tomatoes, which also showed that honeybee pollination could replace PGR pollination to save labor costs (Sabara et al., 2004; Higo et al., 2004; Santos et al., 2009; Cooley and Vallejo-Marín, 2021). The size and shape of some fruits were determined by seed number and distribution (Srivastava and Handa, 2005). This may be related to the fact that tomato fruits have more seeds after bee pollination, and there are reports that the weight of the tomato fruit is positively correlated with the number of seeds (Zhang et al., 2022). However, plant growth bioregulator (PGB) (parachlorophenoxy acetic acid) treatment produced bigger-sized but puffy fruits, but there is no significant difference between PGB treatment and bumblebee treatment (Strange, 2015). This may be related to the different components of the two plant growth regulators. Bumblebee pollination and honeybee pollination both significantly increased the sugar content and decreased the pH value of tomatoes than PGR pollination, and bumblebee pollination of tomatoes had the highest sugar content and lowest PH value. Tomato fruits pollinated by bumblebees contained more fructose and glucose and less sucrose, citric acid, and malic acid (Zhang et al., 2022). CPPU treatment can decrease the soluble solids (Mousawinejad et al., 2014). It can be seen that bee pollination can enhance the taste of tomatoes.

Plant endogenous hormones, including auxin, gibberellin, cytokinin, abscisic acid, and ethylene, are important regulatory substances for plant growth and development, and metabolites (Srivastava and Handa, 2005; Quinet et al., 2019; Jia et al., 2024). We found that there were significant differences in the contents of various endogenous hormones in fruits under different pollination methods. In three treatments, the highest IAA content and the lowest GA content in tomato fruit were found in bumblebee pollination, and the highest GA content was in honeybee pollination. It can be inferred that bumblebee pollination and honeybee pollination function is different at the hormone level. Tomato fruits can produce more seeds after pollination by honeybees and bumblebees, while applying CPPU during flowering can induce seedless tomato fruits (Ding et al., 2013). CPPU promoted gibberellin and auxin biosynthesis in tomatoes to regulate fruit development and induce parthenocarpy (Ding et al., 2013). This is also consistent with the CPPU treatment increasing the fruit-setting rate and yield of tomatoes. After pollination and fertilization, auxin and gibberellin are produced in seeds (He and Yamamuro, 2022). IAA is the main auxin in plants. Auxin can promote cell division and expansion, GA can promote cell expansion (Liu et al., 2023), and auxin can affect fruit set and growth in tomatoes in part by enhancing GA biosynthesis (Ding et al., 2013; McAtee et al., 2013; Kumar et al., 2014). Auxin acts prior to gibberellin in tomato fruit development (Jong et al., 2009), with auxins functioning upstream of gibberellins (Serrani et al., 2008). These suggest that auxin and GA are co-regulated in tomato fruit set and fruit development (Fenn and Giovannoni, 2021; Guan et al., 2024). Auxin and GAs act in a similar way during fruit set in dry fruits (Kumar et al., 2014). When cell expansion ends, the fruit has reached its final size and then enters the maturation stage (Gillaspy et al., 1993). This is consistent with the result that the fruit after bumblebee pollination treatment is larger than that after honeybee and PGR treatments. The contents of ABA, ZT, and iPA in PGR (CPPU) treatment were the highest among the three treatments. The content of ABA in bee pollination treatments was lower than that in PGR treatment. Evidence suggests that gibberellins, auxins, and cytokinins promote plant fruit set and growth, while abscisic acid and ethylene impede plant growth (Niu et al., 2024) and play important roles as inducers of ripening (Zhang et al., 2009). CPPU treatment significantly upregulated the content of IAA and significantly downregulated the content of ABA in melon and pear (Liu et al., 2023, 2018; Cong et al., 2020), which is not consistent with our study, and it may be related to different species. From the end of fruit growth (cell expansion phase, green) to fruit ripening tomato phase (red), the level of ABA was up (Quinet et al., 2019). In tomato ripening, the auxins and ethylene contents of the tomato were increased, and the GA, Cytokinin (CTK), and ABA contents decreased (Quinet et al., 2019). The contents of IAA and ABA were increased during fruit maturation (Kumar et al., 2014). ZT and iPA belong to cytokinins. Cytokinins are generally associated with delaying senescence (Srivastava and Handa, 2005). This also shows that the maturity of tomatoes pollinated by different methods is different, which still needs further research, as the ethylene content and fruit maturity of tomatoes after bee pollination need to be verified.

The taste of the fruit is determined by basic metabolites, which affect the flavor quality of the fruit (Borsani et al., 2009; Li et al., 2024). In this study, we evaluated the relationship between tomato fruit metabolic composition and three different pollination methods. First, non-targeted metabolomics analysis showed that different pollination methods had a large impact on the metabolic profile. This may be due to the more timely pollination of bees, better fertilization effect, a series of fertilization physiological reactions (producing endogenous hormones) after fertilization, promotion of the transport of nutrients to the ovary, and the rapid development of fruits and seeds, resulting in more commercial and nutritious fruits (Klatt et al., 2013; Bommarco et al., 2012). Tomato aroma quality is strictly ripening-dependent once most of the VOCs are produced from fatty acids, carotenoids, and amino acids in metabolic processes that occur during the ripening process (Tobaruela et al., 2021; Quinet et al., 2019). Our metabolomics results confirm this claim: compared with that in PGR pollination, the metabolism of amino acids, vitamins, sugars, flavor substances, and organic acids with antioxidant physiological effects in honeybee pollination and bumblebee pollination groups was strong. This is in agreement with the results of this study, where bee pollination promoted the sugar content and reduced the fruit pH value, and the results of other works of literature on sugar content, acidity, and VOCs (Zhang et al., 2022). In contrast, exogenous CPPU significantly affected fruit size, but not fruit sugar content, titratable acids, and vitamin C contents (Vc) (Mousawinejad et al., 2014). It shows that the fruit pollinated by bees is richer in nutrients and has better flavor (Zhang et al., 2022). This explained that bee pollination could increase sugar content, solid acid ratio, Vc content, and fruit volatiles and reduce acidity, which were related to nutrition and flavor. In honeybee vs. PGR and bumblebee vs. PGR, phenylpropanoid biosynthesis, flavonoid biosynthesis, and stilbenoid, diarylheptanoid and gingerol biosynthesis were mainly significantly enriched. Phenylpropanoids, including caffeic acid, ferulic acid and benzoic acid derivatives, play an important role in plant defense (Isah, 2019; Oliva et al., 2021). Neochlorogenic acid, caffeic acid, chlorogenic acid, ferulic acid, etc., which have antioxidant and antibacterial functions (Osorio et al., 2020; Tang et al., 2022), were significantly more metabolized in bee pollination treatment than in PGR treatment. It can be inferred that the tomato fruit after bee pollination is more favorable to resist diseases. Tomato metabolites pollinated by honeybees and bumblebees are very rich but only have five differential metabolites, and there are differences in endogenous hormone levels, which suggest that honeybee pollination and bumblebee pollination have the same effect on improving tomato flavor by different pathways.

## 5 Conclusion

Here, honeybee pollination, bumblebee pollination, and PGR pollination can improve the fruit-setting rate of tomatoes, and bumblebee pollination can significantly increase the single fruit and fruit size. Honeybee pollination and bumblebee pollination can improve the sugar content of tomatoes and reduce the pH value, while PGR pollination has the opposite effect. Three pollination methods can affect significantly the hormone levels of tomatoes. Honeybee and bumblebee pollination could increase nutrition and flavor, antioxidant and antibacterial metabolites, such as sugar content, reduce acidity, Vc content, fruit volatiles, neochlorogenic acid, caffeic acid, chlorogenic acid, and ferulic acid. These data provide support for uncovering the mechanism of how bee pollination enhances fruit flavor.

However, the nutritional composition of the tomato fruits was not tested in the experiment, which still needs to be improved. The exact molecular mechanism is still unclear and needs further study. We will conduct some studies on the aspects of pollen tube germination and the amount of pollen on the stigma. It is expected that further research may significantly improve our understanding of how pollination methods affect fruit quality.

## Data Availability

The original contributions presented in the study are included in the article/**Supplementary Material**. Further inquiries can be directed to the corresponding author.

## Supplementary material

Supplementary File 1 The total metabolites detected in all treatments.

Supplementary File 2 PCA analysis of all samples.

Supplementary File 3 The percentage of variance and Q2 value in PCA model.

Supplementary File 4 OPLS-DA model parameters.

Supplementary File 5 OPLS-DA model verification plot.

Supplementary File 6 Difference metabolites between three treatments.

Supplementary File 7 The kegg pathway annotation of difference metabolites.
