# Supplementary figures and images for: Effects of different pollination methods on tomato fruits’ quality and metabolism
Source: Front Plant Sci. 2025 Apr 4;16:1560186. doi: 10.3389/fpls.2025.1560186 (PMC12006744; doi:10.3389/fpls.2025.1560186)

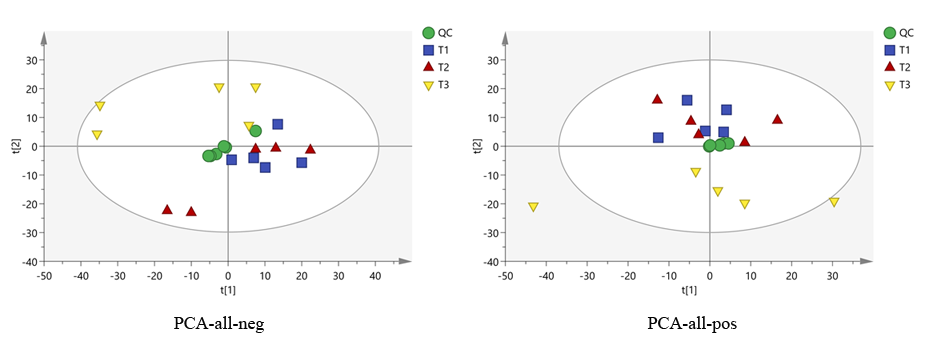

Supplement: Supplementary File 1 — The total metabolites detected in all treatments. [file Image1.tif]

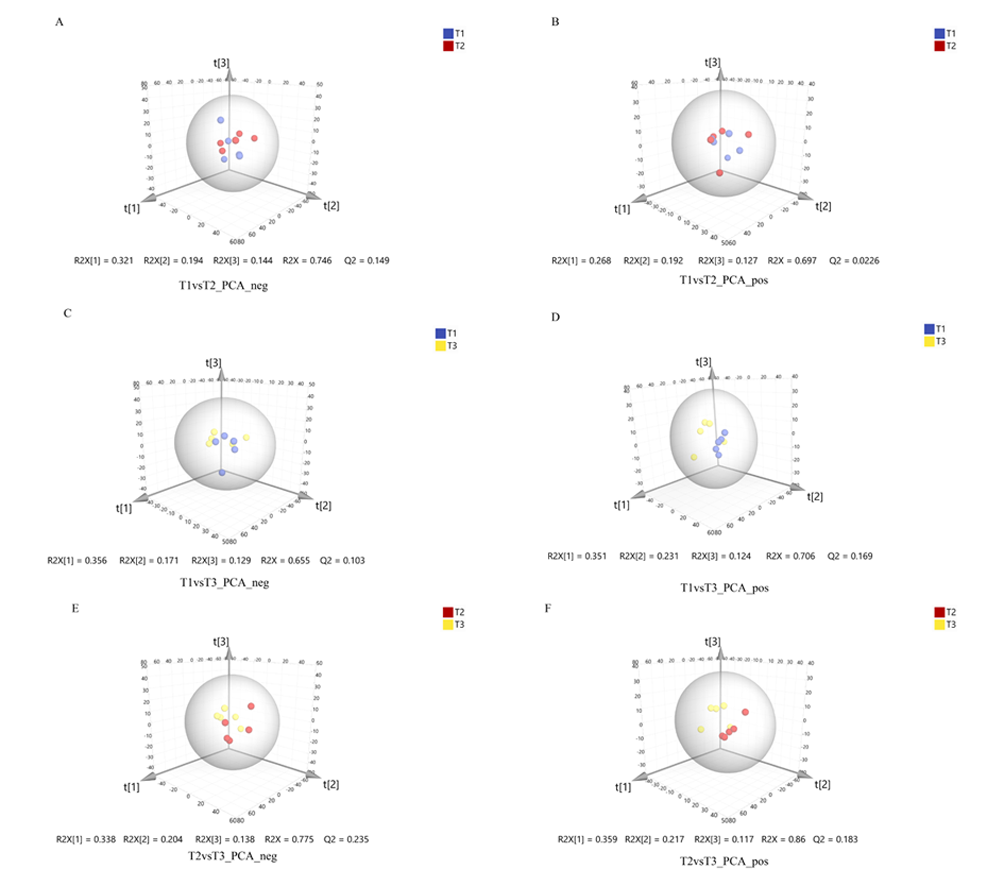

Supplement: Supplementary File 2 — PCA analysis of all samples. [file Image2.tif]

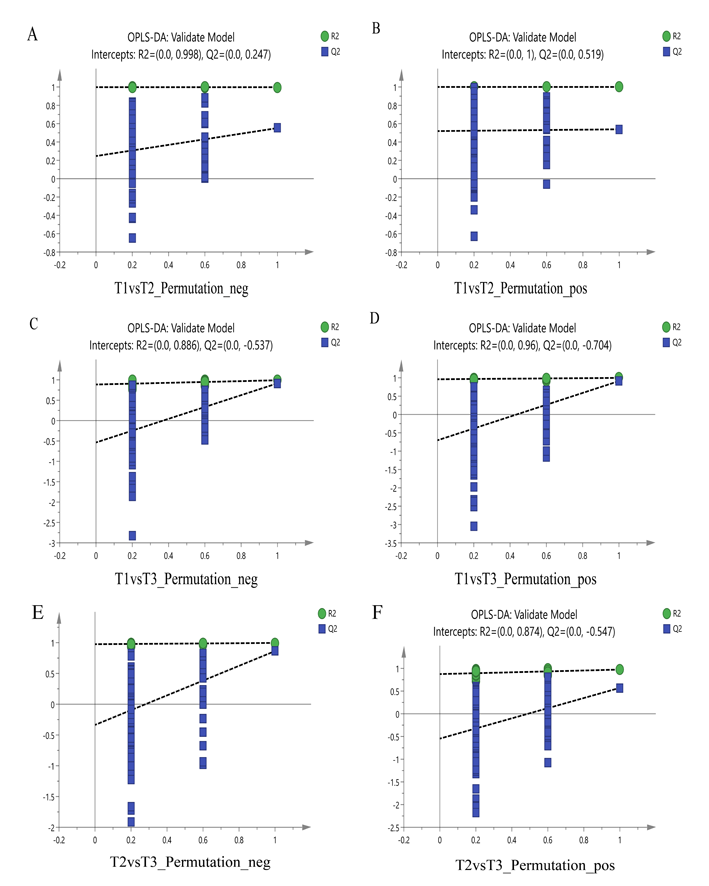

Supplement: Supplementary File 3 — The percentage of variance and Q2 value in PCA model. [file Image3.tif]
